# Mindful Learning: A Case Study of Langerian Mindfulness in Schools

**DOI:** 10.3389/fpsyg.2016.01372

**Published:** 2016-09-12

**Authors:** Chase Davenport, Francesco Pagnini

**Affiliations:** ^1^Department of Education, University of California, Berkeley, BerkeleyCA, USA; ^2^Department of Psychology, Università Cattolica del Sacro CuoreMilan, Italy; ^3^Department of Psychology, Harvard University, CambridgeMA, USA

**Keywords:** mindfulness, mindful education, 21st century skills, creativity

## Abstract

The K-12 classroom applications of mindfulness as developed by Ellen Langer are discussed in a case study of a first-year charter school. Langerian Mindfulness, which is the act of drawing distinctions and noticing novelty, is deeply related to well-being and creativity, yet its impact has yet to be tested at the primary or secondary school level. The objective of the article is to display how Langerian Mindfulness strategies could increase 21st century skills and Social-Emotional Learning in primary classrooms. The New School San Francisco, an inquiry-based, socioeconomically and racially integrated charter school, serves as a model for mindful teaching and learning strategies. It is concluded that when mindful strategies are implemented, students have significant opportunities to exercise the 21st century skills of creativity, collaboration, communication and critical thinking. Langerian Mindfulness is also considered as a tool for increasing Social-Emotional Learning in integrated classrooms. It is recommended that mindful interventions be further investigated in the primary and secondary school context.

## Introduction

Models of educational change have increasingly prioritized the development of creativity, communication, collaboration and critical thinking in 21st century students (Bellanca, 2010). These 21st century skills, often referred to collectively as the “Four C’s,” are considered by many to be prerequisites for students entering a dynamically shifting working world (Kaufman and Beghetto, 2009). How do proponents of the Four C’s propose that teachers plant these skills in their students? Some models call for the need for technological innovation in schools, arguing that computer-based instruction not only widens students’ professional skill base as the workplace becomes increasingly automated but also frees teachers up to focus on relationship building and the development of the Four C’s (Bellanca, 2010). Other educational change agents tout Project-Based Learning as the key to unlocking 21st century skills (Bell, 2010). Advocates of Project-Based Learning maintain that ongoing engagement in collaborative projects expose children to real-life situations in which to practice the Four C’s. In this paper, we will describe how an alternative approach, Langerian Mindfulness (Langer, 1989), could be instrumental in promoting 21st century skills. While Langerian Mindfulness is complementary to the models of educational change described above, we will attempt to highlight how more than 40 years of mindfulness research can improve the social-emotional and academic growth of both students and teachers.

Mindful learning is a divergent and context-dependent approach to ideas. While convergent, narrow thinking plays a vital role in our ability to organize, prioritize and decide, other thought processes expand, rather than condense our ideas (Langer, 1993). Divergent thinking is a mind-expanding process commonly associated with creativity (Nusbaum and Silvia, 2011). To think divergently is to examine an idea by considering its alternatives, creating more possibilities rather than narrowing our focus to one specific answer. When we learn mindfully, we channel this divergent thinking process by considering multiple perspectives in search of multiple solutions to fit multiple contexts (Langer, 1989, 1993).

While there are different approaches to mindfulness (Pagnini and Phillips, 2015), we will refer to the concept developed by Langer (1989), who defines mindfulness as the simple process of noticing new things and drawing novel distinctions. This concept is rooted in the awareness that reality is in constant change. Paying attention of big and subtle changes in reality (either internal or external) forces a person to stay in the present, in the moment. When mindful, people are sensitive to the environment and the context, they create new categories for structuring perception, they welcome novelties, and they present multiple perspectives in problem solving (Langer and Moldoveanu, 2000). This awareness of multiple perspectives helps reducing the need for previously established categories, promoting mind-openness and engagement (Langer, 1992). The opposite of mindfulness, mindlessness, consists in relying on previously established categories. When mindless, one acts as a pre-programmed machine, behaving according to categories created in the past. When that happens, the person is entangled in a single and inflexible perspective, unaware of other possible ways of knowing. Most concepts from social psychology (e.g., stereotypes, prejudices) refer to mindlessness (Langer and Moldoveanu, 2000). According to Langer, mindfulness can be easily taught by inviting people to notice differences and to pay attention to new elements that were not part of the previous schema.

## Mindful Education in Practice

While Langerian mindfulness proved to be a powerful tool for education and learning in undergraduates and high-school students (Langer et al., 1989), there is a lack of reports about the application of her theories in elementary classrooms. In this case study of The New School San Francisco, we explore how Mindfulness has been implemented from the perspectives of both teachers and students. Our exploration of one mindful school will be followed by a discussion of how mindful practices could be applied in other teaching and learning contexts. Our references to “mindful learning,” relate to the mindful practices related to noticing new things and drawing novel distinctions, according to the Langerian mindfulness model (Langer, 1989). Meditation-based practices developed by classroom pioneers such as Snel (2013) have demonstrated powerful effects on children’s focus and social-emotional awareness, but Langerian mindfulness does not incorporate meditation, nor do teachers and students at New School San Francisco. Rather than meditating, New School teachers encourage students to actively notice changes in context and consider situations from multiple perspectives. We will provide explicit examples of these mindful learning techniques throughout this case study.

The New School San Francisco (NSSF) opened its doors in the fall of 2015 as an inquiry-based charter school committed to Social-Emotional Learning. At NSSF, “Inquiry” is a pedagogical process that is “driven by student voice and choice” and that “strongly supports educators to implement student ideas, questions and solutions into the learning progression” (New School San Francisco, 2015) Teachers and administrators at NSSF are encouraged to be responsive to the interests and needs of their students. Many schools have implemented inquiry-based approaches, but NSSF is unique in that it offers inquiry-based strategies to a socioeconomically integrated population. The diverse student body at the New School San Francisco mirrors the demographics of the city of San Francisco. Teachers blend inquiry strategies with practices in Social-Emotional Learning to ensure that students receive an equitable education.

## Celebrating Diverse Perspectives for Social-Emotional Growth

A landmark of a mindful educational process is not only the acceptance, but also the promotion of differences (Langer et al., 1985). Students can increase their social-emotional awareness through immersion in socioeconomically and ethnically diverse communities. Students’ diverse perspectives are not merely tolerated, they are encouraged and celebrated. For example, in the morning class meeting, each student gets a chance to share his or her Weekend News, so that the diverse out-of-school activities take center stage. This is inline with the decades of research suggesting the social-emotional advantages of integrating students from diverse racial and socioeconomic backgrounds (Siegel-Hawley and Frankenberg, 2012). Contrast socioeconomic and ethnic integration with most public and private primary classrooms, in which students are surrounded by classmates who look, speak and act similarly to themselves. Whether a student attends a private school composed of mainly wealthy children or a lower-income public school that attracts primarily lower-income students, the range of perspectives from which (s)he can consider Weekend News may be significantly narrower. Rather than celebrating diverse perspectives, similarity, rather than difference, is more likely to guide thought and action, dulling the children’s capacity to imagine alternatives.

While the students in NSSF explore diverse possibilities, the teachers in this mindful school actively seek alternative perspectives as well. Each classroom is facilitated by two teachers, a Lead Teacher and a Resident Teacher. While the Lead Teachers have an average of 10 years of experience and the average Resident Teacher has spent less than 3 years in the classroom, the relationship in non-hierarchical. The integration of two different approaches to teaching is considered a creative opportunity rather than a compromise. Teachers are given 90 daily minutes to plan and collaborate so that their diverse backgrounds can inform everything from daily lesson plans to classroom behavior plans. For example, the Lead Teachers may recognize that their training in teacher-directed, convergent schooling balances the Resident Teachers’ more divergent, child-directed approach. In planning a lesson on how to add two numbers to make 10, for example, the teachers may discuss the possibility of a direct model by the teacher or a more exploratory, child-centered exploration of groups of 10 materials. Realizing that their two perspectives are compatible, the teachers decide to begin their lesson with a guided exploration of groups of 10, followed by a teacher-led discussion to cement the students’ findings. Research on collaborative teaching has revealed significant benefits for both teachers and students in a variety of schools (Gillespie and Israetel, 2008). At NSSF, the synergy of varied levels of educational experiences and philosophies is what leads to the creative and mindful teaching and learning experience. When confronted with ideas that appear mutually exclusive, mindful teachers recognize and celebrate the possibility of a creative solution that integrates both perspectives.

## Celebrating Diverse Solutions

Students in a mindful context exercise their ability to generate a variety of solutions, rather than converging on one correct answer. In NSSF, for example, students play a game called “Magic Number 10,” in which learners are challenged to think of as many ways as possible ways to add two numbers to make 10. Students create not only different combinations of numbers (8 + 2, 7 + 3) but also different contexts in which the numbers can be applied (10 fingers, 10 students, 10 cookies, 10 dollars).

Contrast this divergent approach with the more convergent method of adding two numbers to make 10. Students would be given the formula “8 + 2 = 10” and may be asked to solve for the answer. Students who learn to add using the convergent method would be likely to apply the addition method only when asked to solve for 8 + 2. The mindful students, however, will readily apply the adding strategies whenever they are reminded of cookies, or dollars or a group of 10 students. Consistent with emerging research on instructional width (Pepkin, 2004), when teachers encourage diverse student ideas into the generation of solutions, students are more likely to use mathematical methods than when the discussion is more narrowly focused on solutions provided by the teacher.

From a teacher’s perspective, the mindful search for alternative solutions could be applied to troubleshooting socially unacceptable classroom behavior. When there is a behavior issue, teachers are challenged to think: “How might this make sense from the students’ perspective?” For example, mindful teachers would fully engage with the perspective of a child who consistently becomes violent, kicking and punching the teachers and students around him. Rather than punishing the student for his behavior, the teachers meet with each other, with the student’s parents and with the school administration to discuss their observations of the student and to consider the variety of ways the behavior might make sense from the student’s perspective. What is the purpose of the student’s violence? How could we teach the child an alternative method of reaching his goal? Could the child be using the violence as a way to avoid the academic work that he loathes? Might the child have language deficits that prevent him from expressing his anger with words, so that he overcompensates by punching and kicking? As the caregiving team for the child develop a range of possibilities to explain the student’s outbursts, each option is considered as the teachers collect data on the antecedents and consequences of the student’s behavior. The teachers begin to understand the student’s current developmental capacity for expressing himself, and can then guide the child up along the developmental spectrum of emotional expression. This approach represents an alternative to a more convergent classroom in which a comprehensive system of rewards and punishments governs behavior. In the convergent case, students are given explicit rules to guide behavior such as, “Keep Your Hands to Yourself” and are either punished or rewarded depending on their adherence to the rule. Not only might this strategy undermine the students’ intrinsic desire to follow the rules by rewarding positive behavior with incentives, it also prevents both teachers and learners from understanding the root of the troubling behaviors, and from growing as a result of this understanding. Teachers learn very little about how students would express themselves in a more realistic context in which peaceful expression of frustration is not followed with a sticker or gold star. Students begin to learn how to please teachers, rather than learning the intricacies of prosocial context-dependent behavior. A child in a convergent classroom may act in the “right” way to get a gold star, but may not realize the contexts in which a hug, or even a shove, may be appropriate.

## Expression of 21st Century Skills

While students at NSSF are not explicitly tested in their development of creativity, communication, collaboration and critical thinking, their mindful learning process provides them with intentional opportunities to exercise these skills. Mindful New School teachers segment their inquiry-based approach into three stages: Exploration, Expression and Exposition. As classrooms devote 4 weeks to each of these three stages, the 12-week learning period evolves into a comprehensive unit of inquiry. During the 12-week period, teachers guide students through activities that require creativity, communication, collaboration and critical thinking.

In the first stage of this learning process, exploration, teachers develop a list of four possible topics in which the students have demonstrated significant interest. The process of generating multiple topics of exploration is mindful in that the teachers are not only encouraging the students to engage in multiple possible areas of study, but also responsively considering topics highly relevant to the students’ specific learning context (Langer, 1993). Over the course of the 4 weeks, the students and teachers explore each topic through “provocations” designed by the teachers to inspire creativity and curiosity in the students. For example, for an exploration of market economies at NSSF, first-grade teachers prompt students to create a factory using clay, blocks and crafting materials. Teachers celebrate diverse processes and products throughout the creative exploration phase. Students exercise both their creative and communicative skills as they are challenged to articulate the significance of their creations and the processes they used to create them.

In the expression phase, teachers choose one especially engaging topic to explore further as students add to their creative and communicative foundation with more complex elements of critical thinking. Teachers expose students to discrete skills and concepts that will help the children consider how the topic of study applies to them personally. For the expression phase of market economies, for example, students are challenged to consider what goods and services do and do not exist in the community. The students would then decide which of these goods or services that they each want to contribute to the community, and which resources will be necessary to execute their community contribution. This expression process is particularly mindful because it encourages students to consider how their learning applies to their specific context. Students critically weigh their observations of their community with their prior knowledge and passions until they develop concrete and personally meaningful goods or services to contribute to their community.

The culminating exhibition phase touches on all four 21st century skills as students collaboratively create a product of their learning and communicate their critical thinking throughout the learning process. Some New School first-graders may team up to create a bread factory after realizing that the community had many factories producing tortillas but few options for producing or consuming bread. Given the collaborative nature of the project, teachers challenge each student to communicate his or her specific roles in the factory-creation process. By explaining their decision to create a bread factory, students critically examine and justify the factors that led to their decision. While fielding questions from peers, teachers and parents, students mindfully consider alternatives to the final products that they created and alternative processes that they could have used along the way.

The process of exploration, expression and exhibition encourages students to consider the various paths that could be taken to a specific solution and the multiple possible solutions to any one problem. That allows students to maintain and cultivate a mindful perspective, that is open and flexible, rather than narrow it down with a pre-prepared outcome. Students grow their capacities to create, communicate, collaborate and think critically while situating their newfound skills and ideas into a personally meaningful learning context.

## Conclusion

Many learning processes utilize a narrowing of perspective at all levels of education (Langer, 1997). A child learning a new language takes diverse stimuli and categorizes experiences into words and phrases. Middle school students clarify their writing by narrowing the scope of their thesis statements. High School students devote countless hours to SAT prep, focusing their attention on finding the right answer for any question. These narrowing thought processes are examples of convergent thinking (Guilford, 1956) which prioritizes speed, accuracy and logic to eliminate possibilities and arrive at a “correct” answer. Teachers in low-income public schools have expressed increased pressure for teachers and learners to converge on a single “correct” answer (Davenport, 2014). Few public schools offer an educational model that prioritizes mindfulness and divergent thinking, which could be more common in private schools.

Alternatively, the application of Langerian mindfulness to pedagogy offers learners ample opportunities to exercise the 21st century skills of creativity, communication, collaboration and critical thinking. Mindful interventions in primary and secondary schools could potentially promote both Social-Emotional Learning and application of mathematical methods. Mindful perspective-taking for students with different socio-cultural backgrounds may promote context-dependent social-emotional awareness. A mindful search for alternative solutions could also promote instructional width, increasing the likelihood that students will apply the mathematical methods being taught. The process of seeking different perspectives celebrates diversity and integration, eventually resulting in improved life satisfaction (Langer, 1989). The classroom application of Langerian Mindfulness is a compelling and potentially paradigm-shifting area of future educational research.

## Author Contributions

FP provided the context for the article. As an associate in Dr. Ellen Langer’s research lab, FP explains why mindful education practices are vital for students in the 21st century. CD contributed the sections on mindful education in practice. As a credentialed primary school teacher and researcher in Dr. Ellen Langer’s research lab, CD discusses how Dr. Langer’s theories are implemented in a particular primary school in San Francisco, CA.

## Conflict of Interest Statement

The authors declare that the research was conducted in the absence of any commercial or financial relationships that could be construed as a potential conflict of interest.
